# Depression in Children and Adolescents with Chronic Kidney Disease—Review of Available Literature

**DOI:** 10.3390/jcm12103554

**Published:** 2023-05-19

**Authors:** Natalia Dryjańska, Katarzyna Kiliś-Pstrusińska

**Affiliations:** 1 Clinical Department of Paediatric Nephrology, University Hospital in Wroclaw, Borowska Street 213, 50-556 Wroclaw, Poland; ndryjanska@usk.wroc.pl; 2 Clinical Department of Paediatric Nephrology, Wroclaw Medical University, Borowska Street 213, 50-556 Wroclaw, Poland

**Keywords:** depression, depressive symptoms, chronic kidney disease, children, adolescents, health-related quality of life, caregiver burden

## Abstract

Depression is a significant health problem gaining increasing relevance, especially among children and adolescents. It is known that the incidence of depression is higher in patients suffering from chronic diseases, such as chronic kidney disease (CKD). This review aims to discuss the prevalence of depression in children and adolescents with CKD and its impact on the quality of life of these patients (HRQoL). The research was conducted using online databases with keywords: depression in children and adolescents, depression and chronic diseases, chronic kidney disease, and health-related quality of life. It was found that the risk for developing depression is higher for adolescents and females, and with the use of negative coping strategies, lack of caregiver nurturance, and poor socioeconomic status. In patients with pediatric CKD, the stage of the disease, age of CKD diagnosis, and type of treatment were found to significantly impact HRQoL and contribute to caregiver burden. Depression was more commonly found in children suffering from CKD. It causes significant mental distress to the child and contributes to the caregiver’s burden. Screening for depression among CKD patients is advised. In depressed patients, transdiagnostic tools should be used to alleviate some of the symptoms. In children at risk of developing depression, preventative strategies should be considered.

## 1. Introduction

Depression is a common and serious health problem affecting adults and children. It is also the most common mental health concern in youth, alongside generalized anxiety disorder [1,2].

According to the Diagnostic and Statistical Manual of Mental Disorders (DSM-5) criteria for children and adolescents, depression (major depressive disorder; MDD) is characterized by a combination of depressed mood or loss of interest or pleasure lasting most of the day, nearly every day for 2 weeks or more. It must be accompanied by four or more additional symptoms and cause clinically significant distress or impairment [3]. MDD is a part of a major group called depressive disorders, also including disruptive mood dysregulation disorder, major depressive episode, persistent depressive disorder (dysthymia), premenstrual dysphoric disorder, and depressive disorder due to another medical condition. Children suffering from depression have a higher incidence of other mental health disorders and health conditions. They also experience difficulties in school and social settings. Self-harm and suicide are one of the most serious consequences of depression, with suicide being the leading cause of death in adolescents aged 15–19. Even more concerning, the rate of mental health disorders, including depression, has risen significantly in recent years [4].

It is generally recognized that depression is more common among those with chronic somatic health problems [5,6]. Depression in the presence of a chronic condition not only impairs emotional and cognitive functioning but also influences how a disease is perceived by the patient and whether they will adhere to medical recommendations, pharmacotherapy, and control visits.

Chronic kidney disease (CKD), especially in its end stage (end-stage renal disease; ESRD) significantly strains the child’s mental well-being [7].

According to the Kidney Disease: Improving Global Outcomes (KDIGO) guidelines, CKD is defined as structural and/or functional abnormalities of the kidney lasting for 3 months or longer, with implications to health [8]. Functional damage is usually characterized by a persistent reduction of the glomerular filtration rate (GFR), the presence of elevated urine protein secretion, or both. Different stages of CKD are defined according to the risk of progression of the disease. In adults, the staging is based on the cause of the disease, GFR category, and albuminuria category (CGA staging). In children with CKD however, as knowledge of the risk of progression or outcomes is still lacking, the staging for clinical care is primarily based on GFR. ESRD is defined as a state of permanent loss of kidney function, generally requiring renal replacement therapy (RRT) [8].

Primary causes of CKD in children consist of congenital abnormalities of the kidney and urinary tract (CAKUT), chronic glomerulopathies, and renal ciliopathies. Structural causes of CKD (such as CAKUT) are dominant in younger children, while the incidence of glomerular diseases increases significantly in children 12 years old and above [9]. 

CKD, depending on the kidney function and clinical presentation, may be managed with conservative treatment or RRT—either hemodialysis (HD), peritoneal dialysis (PD), or kidney transplant (KTx) [8,10,11,12].

In the adult population, the prevalence of depression in patients with CKD is estimated to be even greater than in patients with other chronic illnesses [5]. Patients with chronic kidney disease suffer from a variety of changes that may greatly impact their health-related quality of life (HRQoL): frequent hospitalizations, strict pharmacotherapy schedule, restrictive diets, and limited fluid intake; finally, strenuous regimens following renal replacement therapy—all these factors have an impact on their mental health and may contribute to the development of depression.

This review aims to discuss the prevalence of depression and depressive symptoms in children and adolescents with CKD and their impact on the quality of life of these patients. The second aim of our study was to identify major gaps in current knowledge and possible areas for future clinical research.

## 2. Materials and Methods

**Figure 1:**
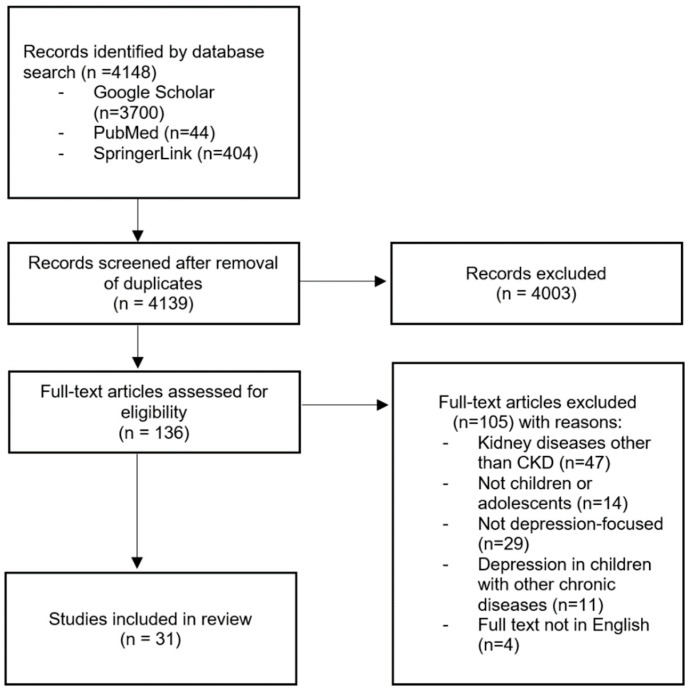
PRISMA diagram of the review process.

For this study, literature from the period of 2000 to 2022 was selected using data collected from three digital databases (PubMed, SpringerLink, and Google Scholar) and in accordance with PRISMA guidelines [13]. An electronic search was conducted twice per database. Keywords used were: depression in children and adolescents, depression and chronic diseases, chronic kidney disease, and health-related quality of life. The search was limited to articles written in English. The initial search yielded 4148 records. After the screening, 136 full-text articles were found. Out of this group, 31 articles consistent with the aforementioned keywords and focusing on depression, in either healthy or CKD children, were selected (Figure 1). 

These included 13 original articles, 9 literature reviews, 7 clinical guidelines articles, and 2 meta-analyses. The majority of articles were published in the years 2016–2021. Out of the 13 original articles, there were 9 major papers that analyzed various aspects of depression (and anxiety) in children and how it impacts pediatric CKD patients, using standardized forms and questionnaires, i.e., Pediatric Quality of Life Inventory 4.0 (PedsQL 4.0) or Children’s Depression Inventory (CDI). We highlighted these studies in Table 1. 

## 3. Results

### 3.1. Depression among Children with CKD

According to the WHO, around 10% of children and adolescents suffer from mental disorders, and of those 3% develop a depressive disorder [21]. Depression seems to be more prevalent in the presence of a chronic disease. Even though survival rates of children with CKD are significantly higher than in the previous decades, issues associated with the disease itself may predispose them to develop depression. The exact prevalence of this psychiatric disorder in pediatric CKD patients varies (10–35%) depending on the progression of CKD (KTx, dialysis, or pre-ESRD) and the age of the child (young children or adolescents). In the study conducted by Kogon et al. [17], 7% of children and adolescents with CKD met the study criteria for depression and 5% reported elevated depressive symptoms [17]. 

Depression is often comorbid with anxiety (up to 30%) [18]. This high rate can be explained by an overlap in symptomatology shared between the disorders [22]. It is also often underdiagnosed and remains untreated [23]. This phenomenon is especially dangerous, as even the depressive symptoms that do not meet the diagnostic criteria contribute to significant impairment in the daily functioning of the child [7,23]. 

Important factors that mediate the development of depressive disorders are the child’s age and sex, implementation of various coping strategies, presence of external stressors, socioeconomic factors, and familial and peer support [7,11,18,19,24].

A child’s age plays a significant role, as depressive symptoms are more frequently reported by adolescents [20]. Adolescence is a crucial moment in a child’s mental health, and almost 50% of mental disorders develop at age 14. Adolescence also often marks the time when CKD in previously ill children progresses into ESRD, requiring kidney replacement therapy [21]. The child’s gender is of similar importance, with girls presenting with depressive and anxious symptoms more often than boys [7,18]. Interestingly, due to the most common causes of CKD in children being structural abnormalities of the kidney (such as CAKUT, which are more often found in males), CKD itself presents more often in boys and as such, the abovementioned observation may not apply to this particular population [9].

Coping mechanisms are a way of dealing with different life stressors and regulating their effect on physiological responses. Emotion regulation (positive and negative) is a primary coping strategy that has been studied in accordance with the development of depressive/anxious symptoms [19]. It is defined as an extrinsic and intrinsic process responsible for monitoring, evaluating, and modifying emotional reactions to achieve one’s goal. When the child perceives the situation as uncontrollable, emotion regulation processes are initiated; however, if the effort is ineffective, it may lead to a vicious cycle, causing increasing psychological distress and further poor attempts at emotion regulation. Deficits in this particular area were associated with anxious and depressive disorders [18]. 

Self-esteem is defined as an individual’s global evaluation of his/her worth as a person. High self-esteem serves a protective role in the development of mental health problems. This trait, however, is known to be unstable during adolescence. In a study performed by Martinsen et al. [24], it was shown that the decline in self-esteem increases the risk of depression [24].

Parental support in the prevention of depression was also studied. It was shown that a safe, supportive environment with engaged and responsive caregivers builds resilience and secure attachment in children—important factors playing a protective role against major life stressors, such as the presence of chronic disease [25,26,27]. Children with chronic diseases are especially dependent on parental support in many areas of their lives. Parents of pediatric CKD patients, to improve the quality of care, adapt the caregiver role in addition to their parental role. This process is especially important as the quality of care has been shown to be a vital determining factor of outcomes for children with CKD. 

In such a situation, however, caregiver tension may be present. Parents tend to prioritize the supervision of their child’s needs to the detriment of their usual lifestyle and career. This impacts the relationship between the child and the parent (often putting the child’s physiological health over their mental well-being) and other family members [15,19]. Family history of kidney diseases should also be considered when analyzing parental (and family) support. Certain causes of CKD, such as autosomal dominant polycystic kidney disease (ADPKD), are hereditary—which often means that not only the child but also the parent and/or other family members struggle with CKD. If the person affected also fills the role of the primary caregiver (PC), they are particularly susceptible to developing mental health issues, such as depression.

Low socioeconomic status (SES) is generally associated with a high incidence of developing psychiatric disorders, more disability, and poorer access to health care [28,29]. Children at high economic risk were more likely to exhibit depressive symptomatology than those at low economic risk. In a study published by Graham et al. [26], it was indicated that the influence of low SES on depressive symptomatology can be ‘buffered’ through the secure attachment of children to their parents [26]. The results of that study confirmed that economic risk was associated with depressive symptoms only among insecurely attached children. 

It is important to note that with CKD come myriad different factors that can cause severe psychological distress, such as the feeling of loss of a previous life, missing out on school and social activities, dependence on medications, and restrictive diet and fluid intake regimens. Pediatric CKD patients may struggle with low self-esteem associated with self-image issues (short stature due to growth restriction, scars after surgical procedures, presence of medical appliances such as dialysis catheters, etc.).

Frequent hospitalizations and transportation (i.e., to a hemodialysis center for children with ESRD) make it difficult for children and adolescents to socialize and spend extracurricular time with peers. This leads to isolation and can affect mental well-being even further [11,19]. Grieving the loss of a previous life, and with it—certain levels of wellness and independence—also plays a substantial role. In a study performed on a sample of 131 adult ESRD patients, it was shown that perception of loss was the strongest predictor of depression [5]. Unfortunately, no such studies were found concerning children and adolescents with CKD.

Adherence to strict diets and fluid intake restrictions improves survival rates in patients with CKD. However, studies on adults have shown that depression may have a negative impact on that [4]. In such a situation, a vicious cycle is formed, where chronic disease-induced depression causes non-adherence that in turn worsens the outcomes of CKD, having even more impact on mental health. Chronically ill children and adolescents are known to struggle with adherence, especially in the presence of emotional, social, family, or mental health problems [30].

### 3.2. Health-Related Quality of Life (HRQoL) in Children with CKD

HRQoL is described as an individual’s perceived physical and mental health over time. This functional domain usually suffers when patients are facing mental problems as children with elevated levels of depressive symptoms report lower levels of quality of life and self-esteem compared to normative samples, with girls and older children reporting the lowest levels [16,24].

CKD seems to be even more detrimental to HRQoL. In the study conducted by Gerson et al. [14], it was found that parent- and child-reported ratings in the physical, school, social, and emotional domains were significantly more impaired when compared to parent and child reports from healthy children after adjusting for a host of sociodemographic and CKD-related factors. This was confirmed in another study [19], where it was shown that patients with CKD scored lower on the PedsQL 4.0 questionnaire than healthy controls.

These findings also differ depending on the stage of the disease, with the biggest detriment to HRQoL being reported in children undergoing hemodialysis or peritoneal dialysis. Patients treated conservatively and those after kidney transplantation scored similarly and showed slightly better HRQoL ratings [19].

Additionally, it was also noted that better HRQoL scores were reported in children with longer disease duration and older age—possibly secondary to having a longer time to adapt to the disease [11].

Studies are showing that medication regimen is an important factor affecting the quality of life of children with CKD; with the number of unique medications having a more negative impact than the volume of medications or amount of administrations per week [31]. This was especially apparent in children < 8 years old, as opposed to older children and adolescents.

CKD potentially affects the quality of life of the child’s caregivers as well, especially the parent that assesses the role of a PC. Parental schooling and socioeconomic status were also linked to the child’s and PC’s quality of life, with low socioeconomic status and schooling associated with lower HRQoL [11].

## 4. Conclusions and Future Directions for Studies on Depression in CKD Children and Adolescents

Various studies have shown that depression is common in children and adolescents with pediatric CKD and is associated with worse HRQoL and disease outcomes. The more advanced CKD is, the greater the impact it has on the functioning of the child, and the higher the risk posed of developing depressive symptoms. 

These issues, however, require further research. The number of studies on depression in CKD children and adolescents is limited, with most of the articles having been published in recent years. The majority of studies on depression/anxiety and HRQoL in CKD children focus on ESRD, with less pressure being put on patients with mild to moderate CKD.

This shows that the problem has been taken into consideration, yet more action needs to be taken in order to properly address it. Even though recent advancement in medical knowledge and quality of care has been shown to have a significant positive impact on patient survival, it is not always associated with improvement in HRQoL [17]. It is one of the goals of contemporary medicine to not only prolong life but mitigate the negative effects disease has on patients and their caregivers’ lives [11]. 

In light of these findings, close cooperation between pediatric nephrologists and mental health institution professionals is advised. Guidelines for the early detection of depressive disorders should be established, and using standardized and approved questionnaires, such as PedsQL 4.0 or Maria Kovacs’ Children Depression Inventory (CDI) [32] will help to quickly determine whether a child presents with anxious or depressive symptoms [23]. The use of such tools would also be helpful for assessing the effectiveness of the treatment.

It seems crucial to establish a certain ‘phenotype’ of a CKD child with a high risk for developing depression. Taking into consideration factors such as the age at which the child was diagnosed with CKD, the overall course of the disease, current GFR category, the age of RRT implementation and the type of treatment, positive family history for CKD, and the underlying cause of CKD (studies have shown that CKD caused by CAKUT is usually characterized by slower progression towards ESRD compared to that caused by glomerulopathy [9]). Such action would increase the vigilance of health professionals, and allow early intervention and the implementation of preventative strategies. Future research should also include an analysis of the non-CKD associated factors influencing the development of depressive disorders: a child’s age and sex, socioeconomic factors, familial and peer support, presence of external stressors, and implementation of various coping strategies; searching for more factors may also prove to be valuable. 

Previous publications indicate certain strategies that may prove important in prevention and coping with depression [5,18,19,24,33]. These include:

− Boosting and upholding self-esteem right after CKD diagnosis;
− Creating positive emotion regulation patterns;
− Implementing active coping strategies to alleviate stress related to the disease;
− Looking at the treatment of CKD as a task to be completed, i.e., eliminating or at least reducing the feeling of loss, changing the temporal focus to the present moment and into the future for long-term progress, searching for new ways to find fulfillment in life in the presence of the disease.

Parental support plays a significant role in a child’s mental well-being [19,25]. It seems that parents—or at least a PCs—should be included when implementing these preventative strategies [15,19,25]. However, parents facing CKD in their children are often exposed to the caregivers’ burden, which may put a strain on the parent–child relationship and in turn have a negative influence on the child’s mental state [8,20]. Therefore, it would make sense to identify the right attitude of parents toward their CKD child and help them develop it. 

For children already presenting with depression, to prevent further escalation of depressive symptoms, pharmacotherapy should be considered and adjusted accordingly in order to minimize interactions with other medications, and possible adverse effects on kidney function. Non-pharmacological strategies such as psychological counseling should be encouraged as an integral part of the treatment.

Because of the comorbidity between depressive and anxious symptomatology, transdiagnostic intervention programs should be considered. Effective interventions to prevent depression are available [34]. In one of the studies, cognitive behavioral therapy (CBT)-based EMOTION program has shown potential for long-term reduction of symptoms of anxiety and depressive symptoms in school-aged children [33]. Participating in an intervention might reduce potential suffering both for children and families [18,20,24]. Such interventions targeting emotional symptoms are general in their nature, do not apply to children with CKD, and do not take into account the specificities of the disease and its therapy (including RRT). In the prevention and treatment of depression in CKD children cooperation of nephrologists with psychiatrists, psychologists, and educators seems to be imperative to maintain optimal care and improve adherence to the strict regimens associated with chronic kidney disease. It is also crucial for future children’s well-being, as they become independent adult patients, to be able to withstand further challenges associated with CKD.

## Figures and Tables

**Table 1:** List of major original articles exploring the issue of depression/anxiety and HRQoL in children/adolescents with chronic kidney disease.

Article	Aim(s) of Study	Sample Size	Age of Children (Years)	Methods (Questionnaires Used)	Conclusions
Gerson et al., 2010 [14]	Evaluation of HRQoL in children with pre-ESRD. To study the association between CKD severity and HRQoL. To identify variables associated with poor HRQoL.	Children with CKD *n* = 402	2–16, M = 11, SD = 4	Pediatric Quality of Life Inventory 4.0 (PedsQL 4.0)	Children with mild to moderate CKD reported overall poorer HRQoL and poorer physical, school, emotional, and social functioning.
Kiliś-Pstrusińska et al., 2013 [15]	To analyze psychosocial aspects of CKD in children treated with APD, mainly HRQoL; also establishing levels of caregiver burden of their PC.	Children with ESRD treated with APD, *n* = 41, and their PC	2–18 years, M = 9.24, SD = 5.09	PedSQL, General Health Questionnaire (GHQ-12), Berlin Social Support Scales (BSSS), Caregivers’ Burden Scale (CBS)	PCs rated the HRQoL of their children lower than the patients themselves. The majority of PCs had medium levels of caregivers’ burden.
Kiliś-Pstrusińska et al., 2013 [7]	To investigate levels of anxiety in children with CKD (st. 3 or higher) and to identify factors associated with the presence of that issue.	Children with CKD st. 3 or higher, *n* = 137	group 1: 8–12 years (M = 10.51, SD = 1.56); group 2: 13–18 (M = 15.57, SD = 1.44)	State-trait Anxiety Inventory (STAI), State-trait Anxiety Inventory for Children (STAI-C)	Significantly higher level of anxiety was found in children and adolescents on HD compared to other groups of participants of the same age and Polish population norms. Moreover, in adolescents, a correlation was found between the anxiety state and disease duration.
Lopes et al., 2014 [16]	To find differences between HRQoL of healthy children and children CKD stage 4–5 (and their PC).	I children and PC *n* = 64, C children and PC *n* = 129	2–18 (no information about M or SD was provided).	PedSQL 4.0, Short Form-36 (SF-36)	HRQoL is negatively impacted in patients with CKD stages 4–5. The results suggest an association between worsening HRQoL parameters and inadequate control of recognized therapeutic CKD treatment targets.
Kogon et al., 2016 [17]	Assessment of depressive symptoms in children with CKD and determining association with patient characteristics, intellectual and educational levels, and health-related quality of life.	Children with CKD *n* = 344	6–17, M = 13 (no information about SD was provided).	Children’s Depression Inventory (CDI); PedSQL 4.0, Wechsler’s Abbreviated Scale of Intelligence	7% of children met the criteria for depression; the presence of depressive symptoms was more strongly associated with decreased HRQoL in all of its aspects (also in the parent-proxy). Moreover, CDI was not related to the change in glomerular filtration rate (GFR).
Loevaas et al., 2018 [18]	To estimate the prevalence of depression and/or anxiety depending on strategies of emotion regulation in schoolchildren.	Healthy children with anxiety/depression *n* = 795, I group *n* = 358, C group *n* = 437	8–12 (no information about M or SD was provided).	Mood and Feeling Questionnaire, short form (SMFQ); Multidimensional Anxiety Scale for Children (MASC); Emotion Regulation Checklist (ERC),	The negative association between children’s symptoms of anxiety/depression, and emotion regulation was found.
Clavé et al., 2019 [19]	To describe the QoL of adolescents initiating HD and to determine the factors associated with QoL.	Adolescents initiating HD treatment, *n* = 32	M = 13.9, SD = 2.0	Vécu et Santé Perçue de l’Adolescent et l’Enfant, Kidcope questionnaire	Compared to the French control, index, energy-vitality, relationships with friends, leisure activities and physical well-being scores were significantly lower in the HD population.
Martinsen et al., 2019 [20]	To assess the effect of the EMOTION intervention program on depression and/or anxiety, and its effect on HRQoL and self-esteem of schoolchildren.	Healthy children with anxiety/depression, I *n* = 358, C *n* = 437	8–12, M = 9.64, SD = 0.93	EMOTION questionnaire, Kinder Lebensqualitat Fragebogen (KINDL), Beck youth inventory-II-self-concept scale (BSCY-II)	According to the reports, children in the intervention group self-reported a larger increase in self-reported QoL compared to the control group.
Abrão et al., 2021 [11]	Analyzing the association between generic and disease-specific quality of life and behavior problems in pediatric patients with CKD stage 3 or higher; also the QoL and mental health of the primary caregiver.	Children with CKD st. 3–5 *n* = 80 and their PC	8–18 (no information about M or SD was provided).	PedSQL 4.0, PedsQL ESRD, Child Behavior Checklist (CBCL), Youth Self-Report (YSR)–for children, SF-36 and Mini International Neuropsychiatric Interview (M.I.N.I.)- for PC’s	Children on PD and HD presented lower scores of generic and specific quality of life. A discrepancy was found between patients’ and caregivers’ QoL perceptions—PC’s proxy report showed higher scores than the one reported by the patient.

HRQoL—health-related quality of life; ESRD—end-stage renal disease; CKD—chronic kidney disease; APD—automated peritoneal dialysis; PC—primary caregiver; I—intervention group, C—control group, M—mean, SD—standard deviation.

## Data Availability

Not applicable.
